# Advanced Technique of Unilateral Biportal Endoscopy on Revision Surgery for Recurred Herniated Interverbral Disc: A Technical Note

**DOI:** 10.1155/2024/4095518

**Published:** 2024-09-26

**Authors:** Hun-Chul Kim, Jin Young Lee, Hyeon Guk Cho, Jeong Woo Park, Sang-Ho Han, Young-Il Ko

**Affiliations:** Endoscopic Spine Surgery Center Daechan Hospital, Incheon, Republic of Korea

## Abstract

**Introduction:** Revision surgery in the spine poses considerable challenges due to the presence of scar tissue and structural differences, necessitating careful surgical planning and precise techniques. In this technical note, we focus on lumbar unilateral biportal endoscopy (UBE) for single-level reoperations, outlining principles and methods for handling soft tissue in such cases.

**Materials and Methods:** We reviewed our surgical approach for lumbar reoperations with UBE, emphasizing the importance of meticulous preoperative planning and bone-centered manipulation. Our technique involves utilizing biportal endoscopy for enhanced visualization and employing specific strategies for managing scar tissue, including the “pull-and-cut technique.” We present two illustrative cases to demonstrate the application of our method.

**Results:** The described approach yielded successful outcomes in both cases presented. Case 1 involved a posterior interlaminar approach for a recurrent disc at the L4–5 level, while Case 2 utilized a far lateral approach for recurrent disc herniation at the L4–5 level. Both surgeries were completed with relatively short operation time, minimal blood loss, and immediate improvement in symptoms postoperatively.

**Conclusion:** Lumbar UBE offers a promising option for safe and effective reoperation in spinal surgery. Our technique, emphasizing bone-centered manipulation and specific strategies for scar tissue management, provides excellent visibility and enables precise tissue handling. Overall, UBE facilitates relatively simple and safe reoperations, contributing to improved patient outcomes in the challenging field of spinal surgery.

## 1. Introduction

Since Soliman's publication in 2015, unilateral biportal endoscopic spine (UBE) surgery has become widely used and has developed rapidly in the field of spine surgery [1]. Accordingly, the indications for surgery have also gradually expanded, and surgery using endoscopy is also being performed in spinal reoperations.

Reoperation of the spine is more difficult than virgin surgery due to structural differences from normal tissue in the surgical area and scar tissue adhering to surrounding tissues. In revision cases, some of this scar tissue must be removed to access the lesion. Scar tissue requires careful manipulation because it adheres not only to surrounding bone, muscle, and ligament, but also to nervous tissue such as the dura. In the case of the spine, unlike arthroscopy of the knee, shoulder, etc., a joint space is not formed and the surgery is performed by detaching the muscle from the bone, so a different approach to adhesion is required during reoperation. Also, unlike conventional open surgery, radiofrequency (RF) cautery and shaver are mainly used in UBE, so it is difficult to deal with revision scars with these tools.

In this technical note, we attempt to describe the basic principles and actual surgical methods that can be used during revision surgery for lumbar UBE in two representative approaches, focusing on methods of handling soft tissue.

## 2. Technical Note

In the case of reoperation, especially if the surgery is performed at the same level and using the same approach, careful surgical planning before surgery is very important. The purpose of reoperation must be clear, and if the intervertebral disc protrudes again, the location of the lesion must be accurately recognized before surgery. The excision of the bone performed in the previous surgery must be identified in advance through a computed tomography (CT) scan, and a decision regarding additional excision must be made. If bony decompression was insufficient in the previous surgery, additional excision may be necessary, and additional bone removal may be necessary to access the lesion.

### 2.1. Advantages of Revision Surgery Using Biportal Endoscopy

When using an endoscope, a clear magnified view can be obtained compared to performing surgery with the naked eye or microscope. Therefore, it is advantageous to distinguish between scar tissue and normal tissue. This expanded field of view and excellent ability to distinguish tissues allows for more detailed manipulation of scar and nervous tissues. In addition, compared to uniportal endoscopic surgery, when two portals are used, more precise surgery can be performed because delicate manipulation is possible using a working device with a high degree of freedom of movement while holding the endoscope with one hand and maintaining the field of view.

### 2.2. How to Deal With Scar Tissue

In all endoscopic surgeries, soft tissue manipulation must be performed based on the bony structure to prevent unnecessary tissue damage. In the case of reoperation, scar tissue is formed, so if resection of scar tissue is performed without planning, iatrogenic durotomy and damage to nervous tissue may occur. Therefore, we would like to suggest some principles in endoscopic reoperation of the spine.

1. Always manipulate the soft tissue based on the bony structure.

**Figure 1:**
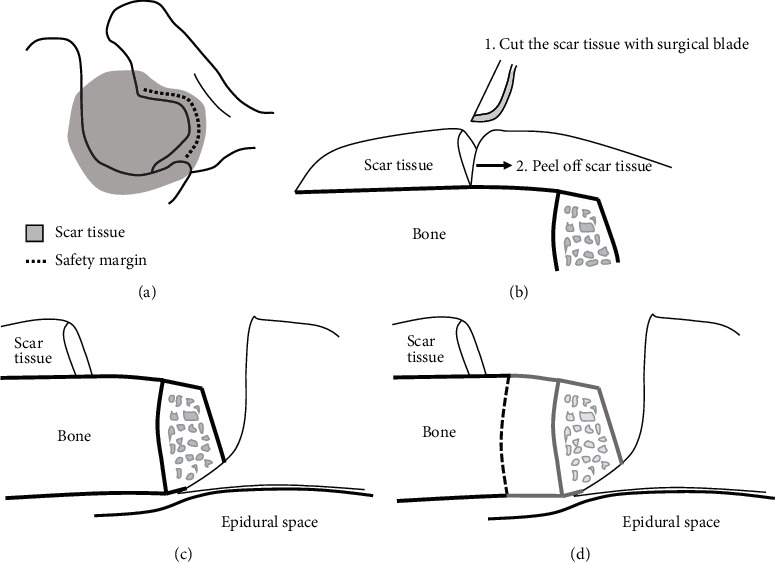
(a) Make the incision above the bone with a surgical blade and peel off the scar tissue. (b) Confirm the safety bone border with a blunt instrument (dotted line). (c) Use a Kerrison punch or curette at the bone margin to carefully create a hole through which the surgical instrument can enter the epidural space. (d) If a space is created inside the adhesion scar tissue, remove a little more bone until normal tissue without adhesion appears and remove the scar tissue covering the epidural space along the bone margin.

No matter how skilled a surgeon is, removing soft tissue without a reference point can cause an iatrogenic injury with a very high probability. The most important principle is to manipulate soft tissue using bone as a landmark. The safety margin made of bone must be checked using a blunt instrument (Figure 1(a)).

2. Cut the scar tissue above the bone.

**Figure 2:**
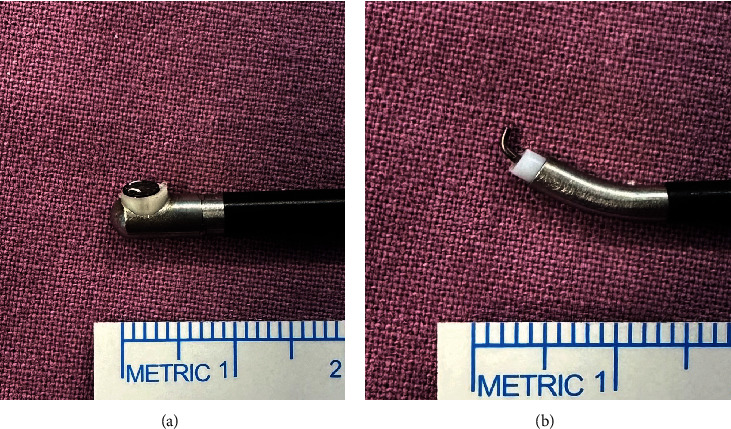
Radiofrequency (RF) electrocautery device. (a) A commonly used blunt tip RF device with a tip bent at 90°. (b) Hook-type RF device.

In the case of endoscopic surgery, a shaver and an RF device are used to remove soft tissue. Because the shaver commonly used has a blunt tip, it is difficult to immediately remove scar tissue that is firmly adhered to the bone. In addition, the tip of the conventional RF device, which is mainly used in arthroscopic orthopedic surgery and endoscopic surgery of the spine, is often blunt and the tip is bent at 90° to reduce damage to the nerve tissue (Figure 2(a)). Additionally, it is not easy to detach the scar tissue that is firmly attached to the bone through cauterization alone. Therefore, sharper type of instrument is needed to remove the scar tissue that is tightly bound to the bone. If the bone is confirmed with a blunt instrument, a sharper instrument can be safely used over that area. When an incision is made into the scar tissue above the bone using a surgical blade, a space is created that can be cauterized using an RF device (Figure 1(b)). Afterwards, the scar tissue is removed by using RF to peel off the scar tissue and using a shaver to clean up the remaining tissue.

3. Approach the epidural space along the bony safety margin.

Once the soft tissue around the lamina and interlamina space has been organized, the next step is to approach the inside of the interlamina space. At this time, the degree of adhesion of the dura and scar tissue cannot be predicted, so surgery must be performed according to precise principles to prevent damage to nerve tissue. First, check the safety margin made of bone around the interlaminar space that was checked earlier. Next, use a Kerrison punch or curette at the bone margin to carefully create a hole through which the surgical instrument can enter the epidural space (Figure 1(c)). If a space is created inside the adhesion scar tissue, remove a little more bone until the normal tissue without adhesion appears and remove the scar tissue covering the epidural space along the bone margin (Figure 1(d)).

4. “Pull-and-cut technique.”

**Figure 3:**
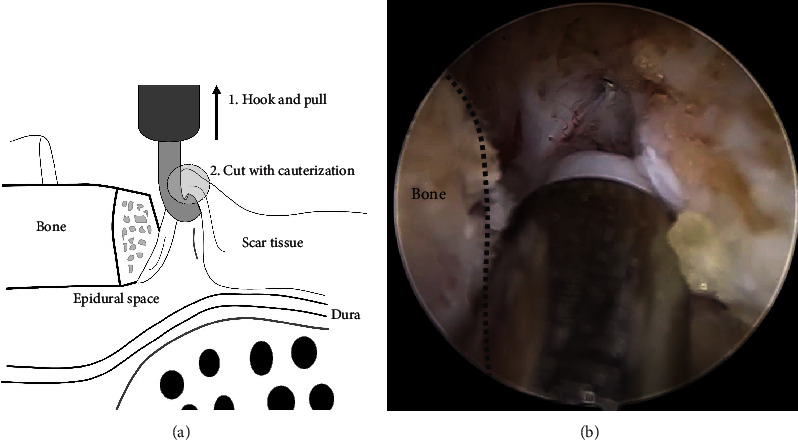
“Pull-and-cut technique.” (a) Using a hook-type RF device, the scar tissue adjacent to the safety margin is hooked, pulled, and cut through cauterization. In this way, damage to the dura can be minimized, and the scar tissue can be quickly removed. (b) Intraoperative endoscopic image.

Within the epidural space, there are often adhesions between existing scar tissue and dura. Because epidural fat is preserved and the dura and scar tissue are rarely clearly distinguished in many cases, delicate manipulation is necessary to avoid incidental dural injury. At this time, we use a hook-type RF device (Figure 2(b)) Using this device, the adhesive tissue is pulled with a hook and cut by performing electrocautery (Figure 3). Through this method, the adhesive tissue can be removed simply, intuitively, and safely and the lesion site can be accessed.

5. Separate the dura and scar tissue only if needed.

Once the epidural space is reached, the outer border of the normal dura can be identified. At this time, if you try to separate the scar tissue and dura to see a more complete shape of the nerve structure, there is a high possibility of causing iatrogenic injury. Additionally, such attempts do not help improve the patient's symptoms and are unnecessary because they are unrelated to the purpose of the surgery. It is sufficient to identify the normal outer margin of the thecal sac, and there is no problem in removing the recurrent disc.

6. Access to disc space: far lateral approach.

In surgery for a recurrent disc in the far lateral approach, it is necessary to check how much bone was removed during the previous surgery and make a plan. If the tip of the superior articular process (SAP) was removed in a previous surgery, select that area as the first target. During surgery, the resected SAP area is palpated with a blunt instrument under a fluoroscopic guide. If it is difficult to confirm on fluoroscopy or the degree of resection is large and difficult to select as a target, palpate the transverse process and isthmus first and find the resected SAP from there. When approaching through that area, it leads to Kambin's triangle, so we can reach the ruptured disc safely. If resection of SAP has not been performed, whether to remove SAP is determined depending on the location of the recurrent lesion, and the facet joint is initially approached as the target.

### 2.3. Case 1: Interlaminar Approach (Supporting Information 1)

**Figure 4:**
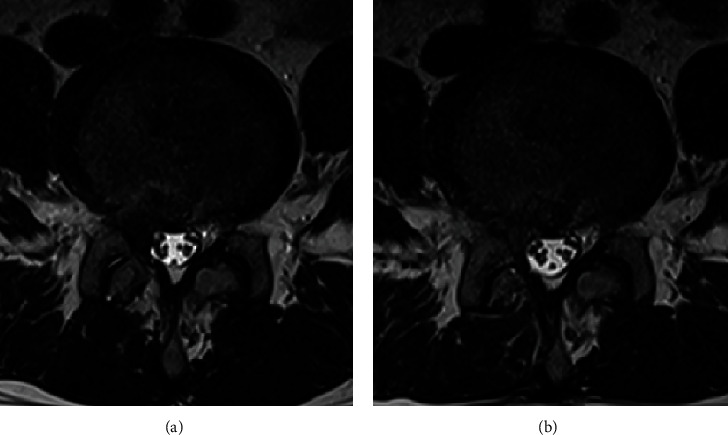
(a) T2-weighted MRI scan of a patient with disc herniation that recurred in the same area after L4–5 disc removal 20 years ago. (b) Postoperative T2-weighted MRI scan.

A 45-year-old male patient who underwent discectomy 20 years ago visited the outpatient clinic due to recurrent radiating pain in the right lower extremity. A recurred disc at the L4–5 level was confirmed on magnetic resonance image (MRI) (Figure 4(a)). Biportal endoscopic surgery was performed using the technique described above using right-side posterior interlaminar approach. The surgery took a total of 25 min, and symptoms improved immediately after surgery. MRI scan performed after surgery confirmed that the herniated disc was successfully removed (Figure 4(b)).

### 2.4. Case 2: Far Lateral Approach (Supporting Information 2)

**Figure 5:**
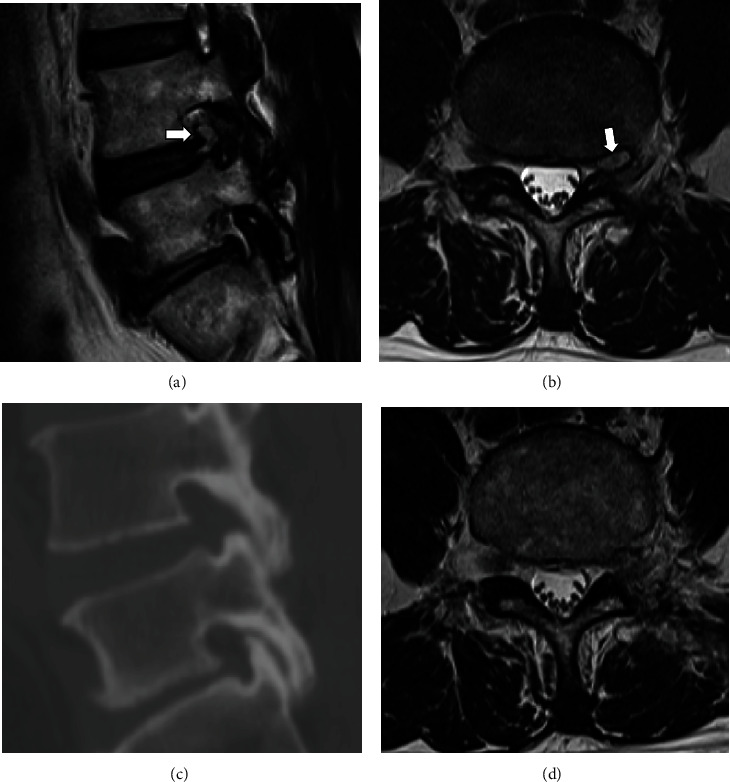
Preoperative (a) sagittal, (b) axial T2-weighted MRI of disc herniation (white arrow) that recurred in the same area 3 months after decompression through a far lateral approach. (c) Preoperative CT scan determines the extent of bone resection performed in the previous surgery. (d) Postoperative MRI confirm that the herniated disc has been removed.

A 59-year-old male patient underwent UBE surgery using a far lateral approach for left L4–5 disc herniation 4 months ago. Symptoms improved after surgery, but radiating pain in the left lower extremity recurred 3 months after surgery, and recurred disc herniation was confirmed on MRI (Figures 5(a) and 5(b)). A left-side far lateral approach was performed using the same incision to remove the recurrent disc lesion. The extent of SAP resection performed during the previous surgery was determined using a preoperative CT scan (Figure 5(c)). The surgery took a total of 30 min, and the radiating pain in the left lower extremity improved immediately after surgery. Postoperative MRI showed that the foraminal disc herniation was removed (Figure 5(d)).

## 3. Discussion

Over the past decade, UBE has significantly transformed the treatment paradigm for spinal disorders. As UBE surgeries increase, revision surgeries using UBE are also increasing. Typical causes of reoperation include postoperative hematoma, incomplete decompression, and recurrence of intervertebral disc herniation. In the case of lumbar disc herniation, the recurrence rate after discectomy has been reported to vary from 2% to 25%. Risk factors for disc recurrence are known to be smoking, obesity, and diabetes, and surgical factors include larger annular defects and removal of an insufficient amount of disc [2, 3].

Many surgeons experience difficulties performing UBE surgery on patients who are not virgins but have had previous surgery. The reason for this is the difference from the existing anatomical structure and the scar tissue that is strongly adherent to the surrounding tissue. Due to the fear of iatrogenic nerve injury that may occur due to the possibility of adhesion to nerve tissue, the surgery time takes longer than before and sometimes the surgery is incomplete. Therefore, when performing such surgery, sufficient experience is required, and precise principles and tissue handling are required.

The biggest advantage of UBE is the expanded field of view obtained because the camera is directly in close contact with the tissue. The continuous flow of water, with 30 to 50 mmHg, removes debridement or microbleeds that obstruct vision, allowing the lesion to be encountered without a suction device that obstructs vision. Additionally, the pressure of the water pushes the dura towards the far side, creating a work space. Unlike scar tissue, which is pale and has no fiber direction, normal tissue is pinkish in the case of muscle, and ligaments also have fiber direction, so they can be distinguished under an enlarged view using an endoscope. This high level of differentiation allows for safe and accurate surgery.

RF electrocautery used in spine surgery is largely divided into monopolar devices and bipolar devices. In the case of monopolar devices, the degree of tissue damage is high because heat is applied directly to the tissue, while in the case of bipolar devices, heat is not applied directly to the tissue [4]. All RF devices used during UBE surgery are bipolar type. Therefore, when using the hook-type RF device described above, surgery can be performed relatively safely even near the nerve tissue.

The UBE surgical area is gradually expanding. In addition to the commonly performed lumbar and cervical foraminotomy, surgery using endoscopy for thoracic spine lesions has been reported [5]. Additionally, the area is expanding to include infections and tumors [6–8]. This is the same in the area of spine reoperation. In a comparative study between primary lumbar discectomy and revision lumbar discectomy using UBE, Kang et al. reported noninferior results in revision surgery compared to primary surgery [9].

The soft tissue management techniques mentioned in this study are basic principles that can be used not only in recurrent disc surgery but also applied to other levels, such as the cervical and thoracic spine, or to other types of reoperations, such as spinal fusion after disc removal.

## 4. Conclusion

UBE can be a good option to perform safe and delicate surgery with low blood loss and excellent visibility in the spinal reoperation area. Reoperation can be performed relatively safely and simply using a bone-centered approach and the principles mentioned in this technical note.

## Data Availability

There is no further relevant data outside what was described in this case report. The identity of the subject of this case report will remain anonymous to observe patient confidentiality.

## Supporting Information

Supporting Information 1 Video S1 (right-side interlaminar approach, Case 1). Using a shaver with a blunt tip, the soft tissue is cleared at the superficial level of the safety bone margin, preconfirmed. Next, scar tissue is identified and partially incised above the bone using a surgical blade. Following this, the scar tissue is removed using Kerrison punch and shaver. Subsequently, a curette is used to create a gap between the bone and scar tissue. It is crucial that the sharp end of the curette is always directed towards the bone, while constantly touching the bone during manipulation. Once the gap is created, the bone is partially shaved using a burr to confirm the intact ligamentum flavum and to verify the passage to the epidural space. Then, a hook-type RF device is utilized for the “pull-and-cut technique” to excise the scar tissue and secure space to the epidural space. After ensuring sufficient space, a Kerrison punch is used to partially excise the soft tissue and bone to further expand the space. Subsequently, a probe bent at a 90° angle is used to confirm the herniated disc, and after retracting the root partially, a pituitary forceps is employed to remove the herniated disc.

Supporting Information 2 Video S2 (left-side far lateral approach, Case 2). Prior to surgery, the degree of resection of the SAP tip is confirmed via CT scan, and any remaining bone is identified using an instrument with a blunt end. Next, the surrounding scar tissue is identified, and incisions are made above the bone using a surgical blade. Subsequently, the scar tissue is cleared using a shaver, and the gap to the epidural space between the previously resected bone margin and scar tissue is verified. The hook-type RF device is then used to safely excise the scar tissue. Approaching the location of the excised SAP safely leads to Kambin's triangle, allowing secure access to the disc space. The root located cranially is protected using a retractor, and the disc is removed upon accessing the disc space.
